# A preliminary study on the induction of dioestrous ovulation in the mare – a possible method for inducing prolonged luteal phase

**DOI:** 10.1186/1751-0147-48-12

**Published:** 2006-07-26

**Authors:** Ylva Hedberg, Anne-Marie Dalin, Malin Santesson, Hans Kindahl

**Affiliations:** 1 Division of Comparative Reproduction, Obstetrics and Udder Health, Department of Clinical Sciences, P.O. Box 7054, Swedish University of Agricultural Sciences, SE-750 07 Uppsala, Sweden

## Abstract

**Background:**

Strong oestrous symptoms in the mare can cause problems with racing, training and handling. Since long-acting progesterone treatment is not permitted in mares at competition (e.g. according to FEI rules), there is a need for methods to suppress unwanted cyclicity. Spontaneous dioestrous ovulations in the late luteal phase may cause a prolongation of the luteal phase in mares.

**Methods:**

In this preliminary study, in an attempt to induce ovulation during the luteal phase, human chorionic gonadotropin (hCG) (3000 IU) was injected intramuscularly in four mares (experimental group) in the luteal phase when a dioestrous follicle ≥ 30 mm was detected. A fifth mare included in this group was not treated due to no detectable dioestrous follicles ≥ 30 mm. Four control mares were similarly injected with saline. The mares were followed with ultrasound for 72 hours post injection or until ovulation. Blood samples for progesterone analysis were obtained twice weekly for one month and thereafter once weekly for another two to four months.

**Results:**

Three of the hCG-treated mares ovulated within 72 hours after treatment and developed prolonged luteal phases of 58, 68 and 82 days respectively. One treated mare never ovulated after the hCG injection and progesterone levels fell below 3 nmol/l nine days post treatment. Progesterone levels in the control mares were below 3 nmol/l within nine days after saline injection, except for one mare, which developed a spontaneously prolonged luteal phase of 72 days.

**Conclusion:**

HCG treatment may be a method to induce prolonged luteal phases in the mare provided there is a dioestrous follicle ≥ 30 mm that ovulates post-treatment. However, the method needs to be tested on a larger number of mares to be able to draw conclusions regarding its effectiveness.

## Background

According to many mare owners, strong oestrous symptoms result in difficulties with training and handling of the animal [1,2]. One method used earlier was to treat such mares with long-acting progesterone [3,4]. However, this treatment is not permitted for use in racing or competition mares according to many European racing authorities, such as the British Jockey Club [5] and the Swedish Trotting Association (STC) [6], and also according to the rules of the International Federation for Equestrian Sports (FEI) [7]. In addition, in Sweden, short-acting progesterone treatment, such as altrenogest orally, is prohibited for use at competition in riding horses [8] and has a recommended drug withdrawal time of 14 days in Standardbred racehorses (personal communication, Peter Kallings). Another method, the crushing of embryos to achieve a prolonged progesterone phase, has recently been presented [9]; also, the insertion of glass or plastic balls into the uterus has been used to prolong the luteal phase in mares [10,11]. However, some consider these methods to be improper and the use of intrauterine balls is not permitted in Sweden in racing horses [6]. Therefore there is a need for alternative techniques. Mares, in contrast to females of other mammal species, have the ability to ovulate during dioestrus, when serum concentrations of progesterone are high. The frequency of dioestrous ovulations varies according to breed; in Thoroughbreds and Quarter Horses as many as 20 % of ovulations occur during the luteal phase [12], whereas the frequency of dioestrous ovulations is very low in ponies [13]. The follicles that grow and ovulate during dioestrus do not cause oestrous signs, due to the suppressive effects of progesterone on oestrous behaviour [12]. Spontaneous dioestrous ovulations that occur between one to four days prior to luteolysis may cause a prolonged luteal phase, due to the immature corpus luteum being refractory to prostaglandins, as shown by exogenous prostaglandin treatment [14]. A refractory state to endogenous prostaglandin for up to four days after ovulation was also demonstrated by the use of intrauterine saline infusion [15,16]. The normal mean dioestrous length, derived from several different reports, is 14.9 days (range 12.1 to 16.3 days) [17] and in one study, prolonged luteal phases lasted for a mean of 63 days (range 35–96 days) [18]. Daels et al. [19] demonstrated that ovulation induced by human chorionic gonadotropin (hCG) during altrenogest treatment could lead to a prolonged luteal phase (5/6 of the mares that ovulated). The present study was designed to evaluate if hCG-induced ovulation during the late luteal phase in mares will cause a prolonged luteal phase. Both the method of Daels et al. [19] and the method used in the present study would eliminate oestrous behaviour for a period of time due to high endogenous progesterone levels. However, an advantage with the method presented in this paper, as compared to the method of Daels et al. [19], is that exogenous altrenogest treatment is not used.

## Methods

### Animals, management and housing

The experiment was performed in April-September 2004. All procedures were approved by the Ethical Committee for Experimentation with Animals, Sweden. Nine mares weighing between 450 and 550 kg and with an age span of four to 20 years were used in the study. Eight of the mares were Standardbred trotters and one was a Swedish warmblood mare (mare A). The mares were kept at the Division of Comparative Reproduction, Obstetrics and Udder Health, Department of Clinical Sciences and were all owned by the department. From the first week of December 2003, the mares were put on light treatment for 16 hours per day. Prior to the experimental period, the mares had been teased daily with a stallion, which showed that all mares had normal oestrous cycle lengths for at least two cycles before commencing the study (three cycles in all mares but one). In April and May, all mares were stabled indoors on straw in individual loose boxes during the night and let out at pasture during the day (approximately 08.00 to 15.00 h). The mares were fed grass hay (morning, noon and evening) and a small amount of oats, soaked sugar beet and a mineral supplement (afternoon). From the first week of June and after at least one month of experimental study at the department, seven of the mares were transported to pasture 30 km away from the department, where they were kept outdoors 24 hours per day until the end of the study. The mares always had free access to water. One mare (mare T) was moved to pasture after only 15 days of study; she had received hCG, but did not ovulate and showed symptoms of oestrus at the department before being moved.

### Experimental procedure

**Figure 1:**
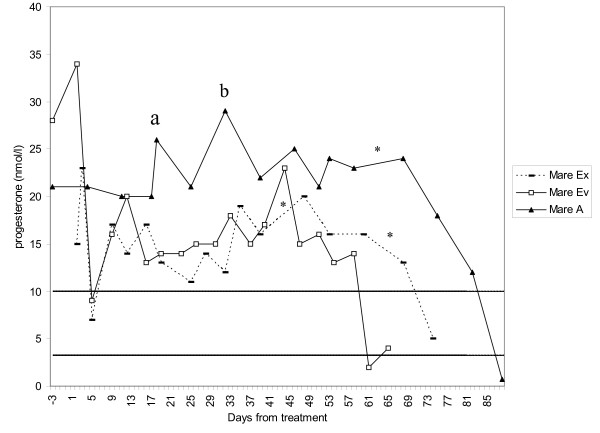
**Progesterone levels in hCG treated mares that ovulated after treatment**. 'a' and 'b' = peaks associated with formation of corpora lutea. * = samples taken 8 or 9 days apart. Dashed lines show the cut-off levels for progesterone samples taken twice weekly (3 nmol/l) or once weekly (10 nmol/l)

The mares were randomly divided into an experimental group (n = 5) and a control group (n = 4). When the mares were at the department, teasing with a stallion was performed daily and oestrous signs were scored as follows: + 1 for tail raise, + 1 for clitorial winking/urination and + 1 for standing in a straddled posture, with behavioural oestrus defined as a score of + 2 or above. When behavioural oestrus was detected, transrectal ultrasound examination (six MHz linear probe, 485 Anser, Pie Medical, Maastricht, Netherlands) of the uterus and ovaries was performed every other day until ovulation was confirmed. Thereafter, from approximately day eight after ovulation in dioestrus, transrectal ultrasound examination was again performed every other day to determine the size of the dioestrous follicles. Three mares (mares Ev, Ma and L) were for technical reasons not examined at oestrus and only followed from day eight after the last oestrous signs were detected; they had a corpus luteum and high progesterone levels at treatment. When a follicle ≥ 30 mm of diameter was detected, 3000 IU of hCG (Pregnyl^®^, Organon AB, Gothenburg, Sweden) or an equal volume of saline (2 ml) was injected intramuscularly. The mares were then followed with ultrasound for up to 72 hours or until ovulation. Thereafter the mares were examined with ultrasound once weekly for three weeks. One mare (mare T) was only examined twice after treatment; she had not ovulated after the hCG injection and showed oestrous signs during teasing eight days after treatment. Starting on the treatment day (hCG or saline), samples for progesterone analysis were obtained from the jugular vein into a heparinised evacuated tube. Thereafter, blood samples for progesterone analysis were taken twice weekly for at least one month and then once weekly for another two to four months. One mare (mare A) was only sampled once a week for the whole duration of the study. This mare, however, was teased daily and examined with ultrasound once or twice per week throughout the study period. In two mares (mares Ex and A) two and one of the samples, respectively, were taken eight or nine days apart (see Fig. 1). All blood samples were immediately centrifuged and the plasma stored at -20°C. Mares were defined as being in luteal phase if progesterone values were above 3 nmol/l [17] in samples taken twice weekly and above 10 nmol/l in samples taken once weekly [20,21]. If no follicles with a size of at least 30 mm were detected in one dioestrous period, the mares were followed as above for, at most, a total of three luteal phases.

### Hormone analysis

The concentration of progesterone in peripheral blood plasma was determined using a solid-phase radioimmunoassay (Coat-a-Count Progesterone, Diagnostic Products Corporation, Los Angeles, USA). The kit was used according to the manufacturer's instructions. The relative cross-reactions of the antibody were 0.9 % with corticosterone and 0.1 % with testosterone. The intra-assay coefficients of variation for progesterone were as follows: 1.4 % at 3.4 nmol/l; 5.2 % at 24.2 nmol/l; 4.1 % at 56.1 nmol/l. The minimal assay sensitivity of progesterone was 0.1 nmol/l.

## Results

The main results are summarised in Table 1. Three of nine mares developed follicles that were above 30 mm (and thus could be treated) in the first luteal phase examined. The remaining mares developed large enough follicles in the second (n = 4), third (n = 1) or in none (n = 1) of the luteal phases studied. Depending on if a mare was treated in the first, second or third dioestrous period after the start of the experiment (due to follicle size), mares were followed for between 90 to 160 days after treatment. Three of the four hCG-treated mares ovulated, resulting in luteal phases, as defined in materials and methods, lasting for 58, 68 and 82 days post-treatment respectively (Table 1). Figure 1 shows their post-treatment progesterone concentrations. These mares were treated between eight and ten days after ovulation was confirmed by ultrasound (every other day) or after last detected oestrous signs (mare Ev), which means their actual luteal phases lasted for at least 69, 77 and 90 days. Two of the hCG-treated mares (mares A and Ev) were never moved to pasture and were therefore teased daily for symptoms of oestrus and mare A examined with ultrasound. The mares showed oestrous signs at days 62 (mare Ev) and 88 (mare A) post treatment and their progesterone levels were then below 3 nmol/l. Mare A had two additional luteal phase ovulations which were associated with two peaks in her progesterone levels (see Fig. 1). The third mare (mare Ex) did not show any symptoms of oestrus during the time she was still kept at the department (37 days). One additional mare in the experimental group (mare O) never developed a dioestrous follicle above 30 mm in any of the three luteal phases studied and was thus never treated. Another mare in this group (mare T) injected with hCG in the third luteal phase studied did not ovulate as a result, although she had a follicle above 30 mm at treatment. This mare showed symptoms of oestrus eight days after the injection had been given. The progesterone level was then low (< 3 nmol/l).

**Table 1:** Summary of results of saline- and hCG-treated mares

**Treatment**	**Mare**	**Cycle no. treated**	**Day after confirmed ovulation**		**Length of luteal phase after treatment**
				
			**treatment was given**	**new ovulation was detected**	
**hCG (3000 IU)**	Mare A	two	8	10	82 days
	Mare Ev	one	10*	13	58 days
	Mare Ex	one	10	13	68 days
	Mare T	three	10	-----	9 days

**Saline**	Mare Ma	one	10*	-----	8 days
	Mare Me	two	7	-----	72 days
	Mare J	two	7	-----	8 days
	Mare L	two	9*	-----	9 days

* Day after last oestrous signs

**Figure 2:**
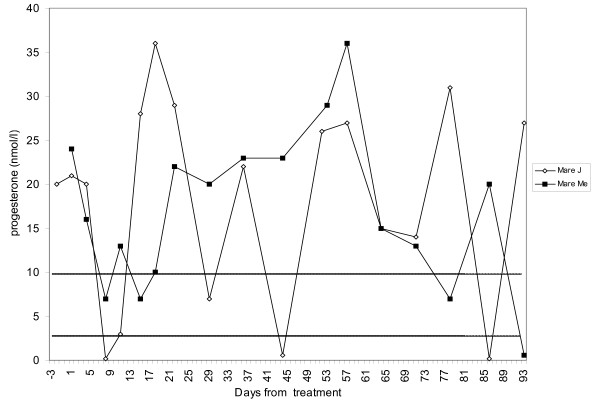
**Progesterone levels after saline treatment in two control mares**. Mare Me developed a spontaneously prolonged luteal phase. Dashed lines show the cut-off levels for progesterone samples taken twice weekly (3 nmol/l) or once weekly (10 nmol/l)

None of the control mares treated with saline ovulated during the luteal phase. In three mares (mares Ma, L and J), progesterone values dropped below 3 nmol/l within nine days post-injection. Two of these mares (mares Ma and L) showed oestrous symptoms in association with the drop in progesterone resulting in dioestrous lengths of 18 and 23 days respectively. Mare J had silent oestrus and ultrasound examination three days after the low progesterone values revealed a new corpus luteum (soft on palpation) and progesterone levels increased to 28 nmol/l four days later. Mare Me never developed a follicle ≥ 30 mm in the first luteal phase studied and got a spontaneous prolongation of this luteal phase. Therefore, she was induced with prostaglandin F_2α _(on day 22 of dioestrus) (1 ml Dinolytic^® ^vet., 5 mg, Orion, Animal Health, Sollentuna, Sweden). She also developed a spontaneously prolonged luteal phase after the saline treatment that lasted for 72 days. Figure 2 shows mare Me's progesterone levels during the study and also the progesterone levels of mare J for comparison with a mare that cycled normally. Mare Me showed no symptoms of oestrus after saline treatment during the time she was kept at the department (28 days).

Progesterone analysis of all of the samples showed that one of the control mares (mare Ma) that came into oestrus after the saline injection thereafter developed a prolonged luteal phase lasting for 76 days.

## Discussion

In the present study, the fact that immature corpora lutea do not respond to the luteolytic effect of endogenous prostaglandin was utilised in an attempt to produce a prolonged luteal phase [15,16,22]. The mares in the hCG treatment group that developed prolonged luteal phases ovulated between days ten and 13 in dioestrus. Thus, the corpora lutea were a maximum of four days old, and presumably refractory, at the time of endogenous prostaglandin release which occurs around days 14 to 17 of dioestrus [22,23]. Failure of the endometrium to release prostaglandin at the time of luteolysis (for example due to chronic endometritis, maternal recognition of pregnancy or for idiopathic reasons) can also cause a prolonged luteal phase in the mare [23-27]. It would have been of interest to measure prostaglandin metabolite levels to rule out that impaired prostaglandin release was not a cause of the prolonged luteal phases. Unfortunately, this was for practical reasons not possible in the present study. Although all of the mares had shown normal cycle lengths prior to the study, one mare (mare Me) developed a prolonged luteal phase in the first cycle studied and also, in the subsequent, saline treated, cycle.

The mares that ovulated as a result of the hCG treatment developed prolonged luteal phases that lasted for approximately two to three months. Other studies have also shown prolonged luteal activity in the mare to last for similar lengths of time [28,29]. It has been proposed that in order for the uterus to produce and release prostaglandin in response to oxytocin (as occurs at luteolysis), a period of low progesterone levels (i.e. oestrus) followed by a period with elevated progesterone concentrations (i.e. dioestrus), is required [27,30,31]. An initial prolongation of the luteal phase, where a drop in progesterone levels does not occur at the expected time, seems to render the uterus less sensitive to oxytocin [27]. This in turn results in impaired prostaglandin synthesis and release and a prolonged luteal phase lasting for up to three months. For unknown reasons, the uterus eventually regains its responsiveness and oxytocin induced prostaglandin production is again possible, finally resulting in luteolysis and oestrus [27,29].

It has been suggested that prolonged luteal activity may be caused by more than one corpora lutea developing in succession rather than by the persistence of a single corpus luteum [32]. In the present study, the luteal phase length was determined by progesterone analysis alone, and therefore it is not known if the corpora lutea developed as a result of the hCG treatment was active throughout the whole prolonged luteal phase or whether subsequent corpora lutea developed. One mare (mare A), however, was examined with ultrasound throughout the study. In that mare, two peaks in the progesterone curve were found to be associated with two ovulations during the luteal phase (in addition to the hCG induced ovulation), which might have contributed to the increased progesterone levels (see Fig. 1).

Of the mares that ovulated as a result of the hCG injection, 100 % (3/3) developed prolonged luteal phases, i.e. 75 % of the total number of hCG- treated mares. Among the control mares, 25 % (1/4) developed a spontaneously prolonged luteal phase in the saline treated cycle (this mare showed prolonged luteal activity twice during the study; see results). In the oestrous cycle following the saline treated cycle, another mare developed a spontaneously prolonged luteal phase. Thus, in total, 2/4 control mares showed prolonged luteal activity. The incidence of so-called spontaneous prolongation of luteal activity in the mare has been reported to be common in some breeds. One study following seven mares (Thoroughbreds and Quarter Horses) over a two-year period reported 12 cases of prolonged luteal phase [29]. Another study has reported an incidence of prolonged luteal phase of only 3.6 % in trotter mares [33].

According to Fortune [34], only about one third of mares have a secondary follicular wave. Other studies, however, show that the majority of mares do have a secondary follicular wave that occurs during dioestrus, which is accompanied by a rise in FSH and can lead to ovulation [18,35]. Breed differences may explain these divergent findings. Studies have demonstrated that Thoroughbreds have a higher incidence of two major waves compared to Standardbreds, Quarter horses and ponies [17,36]. The present study indicates that maximum follicular size during dioestrus may vary within the same animal and that some mares may never develop large follicles (> 30 mm) in the luteal phase. Perhaps using Thoroughbreds as opposed to Standardbreds would have shown different results.

One hCG treated mare with a dioestrous follicle above 30 mm did not ovulate as a result of the treatment. Few studies have been performed on what the optimal follicle size is when inducing ovulation with hCG, although most studies use 35 mm as minimal size when mares are in oestrus, which is also followed in practice [27,37,38]. That the follicle is actively growing, and not in regression, is probably crucial [17]. The reason for selecting a follicle size of 30 mm or above was because it was suspected that few mares would develop very large follicles in dioestrus, which also was confirmed. The follicle of one mare did not ovulate after hCG-treatment, maybe because of the immaturity of the follicle. It has been shown that only follicles with nine to ten granulosa cell layers respond to FSH and continue their growth and that only a few of these follicles are able to ovulate [34]. Oestrogen and androgen production by the follicle seems to be important in its final maturation and it has been demonstrated that follicles developing during the luteal phase produce less oestrogens, which may result in dioestrous follicles being less responsive to hCG [34].

Thus, there are limitations to the usefulness of the present method in practice. Mares may have to be followed for more than one luteal phase before a follicle of suitable size is detected and some mares may never develop large enough follicles. Also, even if the follicle is larger than 30 mm, it is not certain that ovulation will occur. However, modifying the method by using repeated injections of hCG in smaller doses may give better results. Ovulation of follicles as small as 20 mm has been achieved using 200 IU of hCG given daily (seven out of eight pony mares ovulated in a mean of nine days) [39].

## Conclusion

As a method of inducing prolonged luteal phase in the mare, the results of this preliminary study may suggest the use of hCG in the late luteal phase, providing a follicle greater than 30 mm is present and ovulates as a result of the treatment. The method does not involve the use of substances banned for competition purposes and could thus potentially be used for competition mares whose performance is affected by the oestrous cycle. However, to conclude that the method is effective and applicable to practice, it is necessary to repeat the study using a larger number of mares.

## Competing interests

The author(s) declare that they have no competing interests.

## Authors' contributions

All authors participated in the design of the study. YH, MS and AMD examined the mares and participated in sample collection. MS analysed the blood samples. YH and MS drafted the manuscript. AMD and HK conceived of the study, participated in its coordination and helped to draft the manuscript. All authors read and approved the final manuscript.
